# Age Differences in COVID-19 Stress and Social Ties during the COVID-19 Pandemic: Implications for Well-being

**DOI:** 10.1093/geroni/igaa057.3520

**Published:** 2020-12-16

**Authors:** Kira Birditt, Angela Turkelson, Karen Fingerman, Courtney Polenick, Akari Oya

**Affiliations:** 1 University of Michigan, Ann Arbor, Michigan, United States; 2 The University of Texas at Austin, Austin, Texas, United States

## Abstract

The experience of the COVID-19 (coronavirus) pandemic and its implications for well-being may vary widely by age group across the adult lifespan. The purpose of this study was to examine age differences in stress related to the pandemic and social ties, and whether those experiences are linked to well-being. Participants included a total of 645 adults (43% women) ages 18 to 97 (M = 50.8; SD = 17.7) from the May 2020 nationally representative Survey of Consumers. Participants reported the extent to which they felt stress related to the pandemic in the last month as well as social isolation, negative relationship quality, positive relationship quality, and frequent depression, anxiety and rumination in the past week. Results showed that older people reported less COVID-19 related stress, less social isolation, and lower negative relationship quality than younger people. Greater stress, social isolation, and negative relationship quality were associated with poorer well-being and greater social isolation, and negative quality ties exacerbated the effects of stress on well-being. Although many researchers have indicated that older adults may be more vulnerable to COVID-19 related stress and social isolation, this study indicates that young adults may be relatively more vulnerable. Because isolation and negative relationship quality appear to exacerbate the effects of stress, reducing social isolation and negative relations are potential targets for intervention.

